# Esketamine and Hopelessness: Very Short-Term Effects

**DOI:** 10.1192/j.eurpsy.2024.1635

**Published:** 2024-08-27

**Authors:** F. A. Rodríguez Batista, E. E. Morales Castellano, A. M. Morales Rivero, M. Martínez Grimal, S. Trufero Miguel

**Affiliations:** ^1^Department of Psychiatry, Doctor Negrín University Hospital of Gran Canaria; ^2^Department of Psychiatry, Insular University Hospital of Gran Canaria, Las Palmas de Gran Canaria. The Canary Islands, Spain

## Abstract

**Introduction:**

Treatment Resistant Depression is a challenging condition with a poor outcome and limited therapeutic options. Esketamine is the enantiomer of Ketamine and has recently been approved and marketed for treating depression. Questions remain about its short- and long-term benefit, as well as its usefulness in suicide risk. Hopelessness is one of the symptoms most closely associated with suicide risk.

**Objectives:**

The aim of this paper is to evaluate the effect of this drug on hopelessness after one month of treatment with Esketamine.

**Methods:**

The Beck Hopelessness Scale (BHS) was administered to patients receiving Esketamine at the Doctor Negrín University Hospital of Gran Canaria, who provided informed consent and exhibited suicidal ideations and depressive symptoms at the beginning of treatment. This scale was administered before the intranasal administration of Esketamine and after one month of treatment.

**Results:**

Participants (n=5) had an average age of 54,4 years (median 56). We observed variability in the results among the evaluated patients, although the overall trend was a decrease in scores. On average, the patients’ scores decreased from 14,6 to 7,4 points (with a median change from 14 to 8 points).

**Conclusions:**

Hopelessness improved in the BHS after one month of treatment with Esketamine. These results could be of clinical significance. Hopelessness is associated with suicide risk, so we hypothesize that the improvement could have an impact on it. Nevertheless, we must exercise caution with these results: the sample size is small, patients were taking different medications, and they have diverse medical histories.

**Disclosure of Interest:**

None Declared

